# Monitoring and evaluation of an artificial intelligence-enhanced wound care intervention in a rural health network: defining stakeholder expectations and shared priorities

**DOI:** 10.3389/fdgth.2026.1774616

**Published:** 2026-06-15

**Authors:** Ibukun-Oluwa Omolade Abejirinde, Isabelle Choon-Kon-Yune, Rebecca Johnson, Ugonna Ofonagoro, Rebecca Brookham

**Affiliations:** 1 Institute for Better Health, Trillium Health Partners, Mississauga, Ontario, Canada; 2 Dalla Lana School of Public Health, University of Toronto, Toronto, Ontario, Canada; 3 Institute for Health System Solutions and Virtual Care, Women’s College Hospital, Toronto, Ontario, Canada; 4 Office of Research and Innovation Brightshores Health System, Owen Sound, Ontario, Canada

**Keywords:** artificial intelligence, decision-making, evaluation, healthcare, wound care

## Abstract

**Introduction:**

Chronic, hard-to-heal, wounds such as diabetes-related foot ulcers, venous leg ulcers, and pressure injuries, represent a growing global burden, contributing to high morbidity, and increased healthcare costs and demands on clinical capacity. These challenges are amplified in rural and remote settings, where limited resources and availability of services, workforce shortages, and geographical barriers restrict access to specialised wound care. Artificial intelligence (AI)-enabled tools offer potential to enhance wound assessment, standardise documentation, and expand access to wound specialists. However, their real-world implementation in rural environments remains under-evaluated, particularly regarding contextual factors that influence adoption, sustainability, and equitable impact.

**Methods:**

This study aimed to develop an evaluation framework to guide the implementation of Swift Medical's technology across Brightshores, a rural multi-site health system. A literature scan was conducted to identify key performance indicators related to wound care experience and outcomes, which were refined through ongoing implementation partner meetings. To supplement these findings, semi-structured interviews were conducted with frontline staff (*n* = 2) and senior leaders (*n* = 4), focusing on alignment with organizational priorities, anticipated impacts on clinical practice, and potential challenges related to scaling and sustaining the technology.

**Results:**

Findings from the literature scan, interviews, and implementation meetings were triangulated to identify core value propositions and priority outcomes for evaluation. The literature scan provided a foundational set of validated metrics. Interview findings revealed contextual insights regarding technology use in rural care settings, in which three key themes emerged: (1) expectations of the wound care technology; (2) considerations for implementation; and (3) potential barriers to adoption and implementation. Implementation meetings supported feasibility assessment and refinement of indicators within the implementation context. These insights informed the development of a Monitoring and Evaluation framework grounded in the Quintuple Aim, integrating process, outcome, and contextual indicators that reflect the realities of rural health system implementation.

**Discussion:**

By grounding evaluation design in the lived experiences and decision-making needs of those closest to implementation, this work demonstrates how context-sensitive evaluation frameworks can strengthen digital health decision making in rural systems. The findings offer practical guidance for health organizations considering the adoption, sustainability, and scale-up of AI-enabled wound care technologies.

## 1 Introduction

Chronic wounds such as diabetes-related foot ulcers, venous leg ulcers, and pressure injuries amongst others, represent a significant and growing global burden, placing considerable strain on the delivery of health services. The “silent epidemic” of chronic wounds is attributed to an aging population and an increased burden of multi morbidities (1, 2). The global prevalence of chronic wounds is estimated to be 2.21 per 1,000 population (3) and projected to reach 400 million by 2025 (4) with associated healthcare costs up to $18.7 billion USD by 2027 (1). In Canada, the financial burden of wound care is substantial, with costs surpassing $12 billion CAD in 2023, with about a third of this ($4.5 billion) in the province of Ontario alone (5). Unlike acute wounds, which heal within a predictable timeframe, chronic wounds often persist for months or years with adverse compounding effects on patients' quality of life (6, 7).

Hard-to-heal wounds are associated with high morbidity, prolonged hospital stays, higher infection risks, and in severe cases, amputations (8, 9). The management of chronic wounds at scale is further complicated by reliance on hands-on care, involving manual assessments of wound size, depth, presence of drainage, and tissue type to guide treatment decisions (2). In the context of limited human resources with the required wound care expertise, manual assessments can be time consuming and prone to human error and inconsistency, leading to increased risk of slow healing and complications (2).

Despite the growing demand for effective wound management, access to specialised care remains disproportionately distributed, with urban centres benefiting from a high concentration of wound care specialists and advanced treatment modalities (10). In contrast, rural and remote healthcare settings face numerous challenges due to limited resources, workforce shortages, and logistical hurdles due to wide geographical distances (11, 12). Technological innovation is transforming health service delivery worldwide at an unprecedented pace. At the forefront of these, artificial intelligence (AI) and other digital health technologies offer promising solutions to support wound care by enhancing assessment, streamlining treatment plans, supporting remote monitoring, and expanding access to specialist care (13, 14). AI-driven tools, including machine learning algorithms and computer vision, have demonstrated the ability to automate wound measurements, predict healing trajectories, and provide clinical decision support, potentially reducing costs while improving care accessibility and patient outcomes (15). In rural settings, AI-powered point-of-care innovations can thus be a game changer. However, despite its potential, the implementation of AI in rural settings is not without its challenges; researchers have noted the need for adequate training of clinicians and ensuring the reliability of AI systems in resource-limited environments (14, 16).

For example, in their study examining the implementation of EyeArt, an AI-enabled diabetic retinopathy screening tool, in a primary care centre in Norway, Nolan et al. reported several obstacles to initial adoption, including technical limitations, limited staff availability and on-site technical support (17). Similarly, a literature review by Olugboja and Agbakwuru (18), found that AI adoption across rural health systems is frequently hindered by connectivity challenges, inadequate digital infrastructure, and the need for ongoing training to ensure consistent data capture. These issues underscore that without sustained capacity building and infrastructure investment, AI-supported diagnostic tools may struggle to deliver reliable and equitable benefits in rural and remote contexts. Despite growing interest in rural applications of AI, there remains limited understanding of how such tools function in practice across complex rural environments, highlighting the need for evaluation approaches that can capture contextual nuances, identify implementation barriers, and assess equity-relevant impacts.

### 1.1 Setting and implementation context

#### 1.1.1 Brightshores health system

Brightshores Health System (Brightshores) is one of Ontario's largest rural acute care multi-site hospitals, comprising six locations that serve the Grey Bruce region (>8,500 km^2^) with a catchment area of approximately 175,000 residents. Uniquely, the Grey Bruce region has the fastest growing population of adults aged 65–84 years in Ontario (19). Wound prevalence in the region is high and compounded by geographic barriers to care and limited availability of specialised wound care experts. At the time of writing this paper, Brightshores has only one Nurse Wound Specialist who specialises in Skin Wound Ostomy and Continence, and whose role includes inpatient and outpatient care as well as nursing education. This makes them the primary contact for wound care across all six sites of the network and often requires them to travel to different locations (27–70 km away) for in-person wound assessments.

The Brightshores Nurse Wound Specialist is supported by one Nurse Practitioner (NP) in the diabetic foot ulcer (DFU) clinic who triages all referrals to the foot clinic which are largely received by fax. The foot clinic is located at the Owen Sound site of Brightshores and receives outpatients once a week, with the remaining time spent following up on acute cases and ensuring patients receive appropriate treatment. Brightshores leadership has recognised a need for efficient and effective wound management solutions that support the capacity of the small clinical team and reduce the risk of burnout, while enhancing quality and timely patient care.

#### 1.1.2 The advanced artificial wound care network (AAWCN)

**Figure 1:**
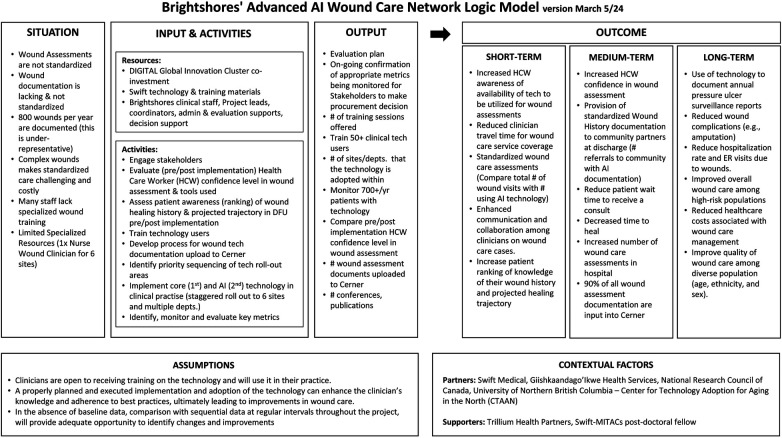
Intervention logic model advanced artificial wound care network.

The AAWCN is a 2-year (initiated in 2023) collaborative multi-partner project in Ontario, Canada, forming a consortium of partners which includes Brightshores Health System, Swift Medical Inc., Giishkaandago'Ikwe Health Services, the National Research Council of Canada, and the Centre for Technology Adoption for Aging in the North at the University of Northern British Columbia. The AAWCN aims to revolutionise the field of wound care, through AI-powered assessment technology and collaborative partnerships, enabling Swift Skin and Wound technology across health systems, including Brightshores. Swift Skin and Wound is a smartphone-based wound imaging and documentation tool that integrates AI-enabled image analysis to support standardised, non-invasive wound assessment. The tool is able to capture wound images and measures, including wound dimensions (e.g., area and depth), tissue composition, and longitudinal healing trajectories, which are displayed through a digital dashboard providing both snapshot and historical views of patients' wound history. These outputs are designed to support clinical documentation, inform care planning, and facilitate communication across care teams. Wound images and associated clinical data are securely stored on access-controlled servers in accordance with organizational policies and privacy legislations and are accessible only to authorised clinical staff for clinical care and approved evaluation purposes. Over the course of 2 years, the AAWCN initiative is implementing and evaluating the ability of Swift Medical's wound care technology to enhance the quality of wound care at the six hospitals within Brightshores Health System. Figure 1 below captures the interventions' logic model and expected short-, medium- and long-term outcomes.

Implementation of Swift Skin and Wound at Brightshores presents both a timely opportunity and need to evaluate the impact of the intervention. However, research has shown that digital health evaluation often misses the opportunity to generate evidence that truly informs decision making by system administrators, funders and health leaders (20–22). Engaging stakeholders who are closely involved in organizational decisions and frontline service delivery can help to identify workflow challenges, resource constraints, and contextual factors that shape how AI tools are integrated into routine practice. Their insights may also help to support the development of evaluations that are aligned with local and organizational priorities and capable of informing long-term planning and resource allocation (23). For evaluation findings to adequately support decision making, the perspectives of those who can influence adoption, sustainability and potential scale up of this AI-technology, must be considered (24).

### 1.2 Objective

The primary objective of this paper is to present insights on the process and outcome of developing the evaluation framework for the implementation of Swift Medical's wound care technology within Brightshores Health System. We gathered the perspectives of frontline staff, healthcare leaders, and implementation partners to define the metrics and measures of success.

## 2 Materials and methods

**Figure 2:**
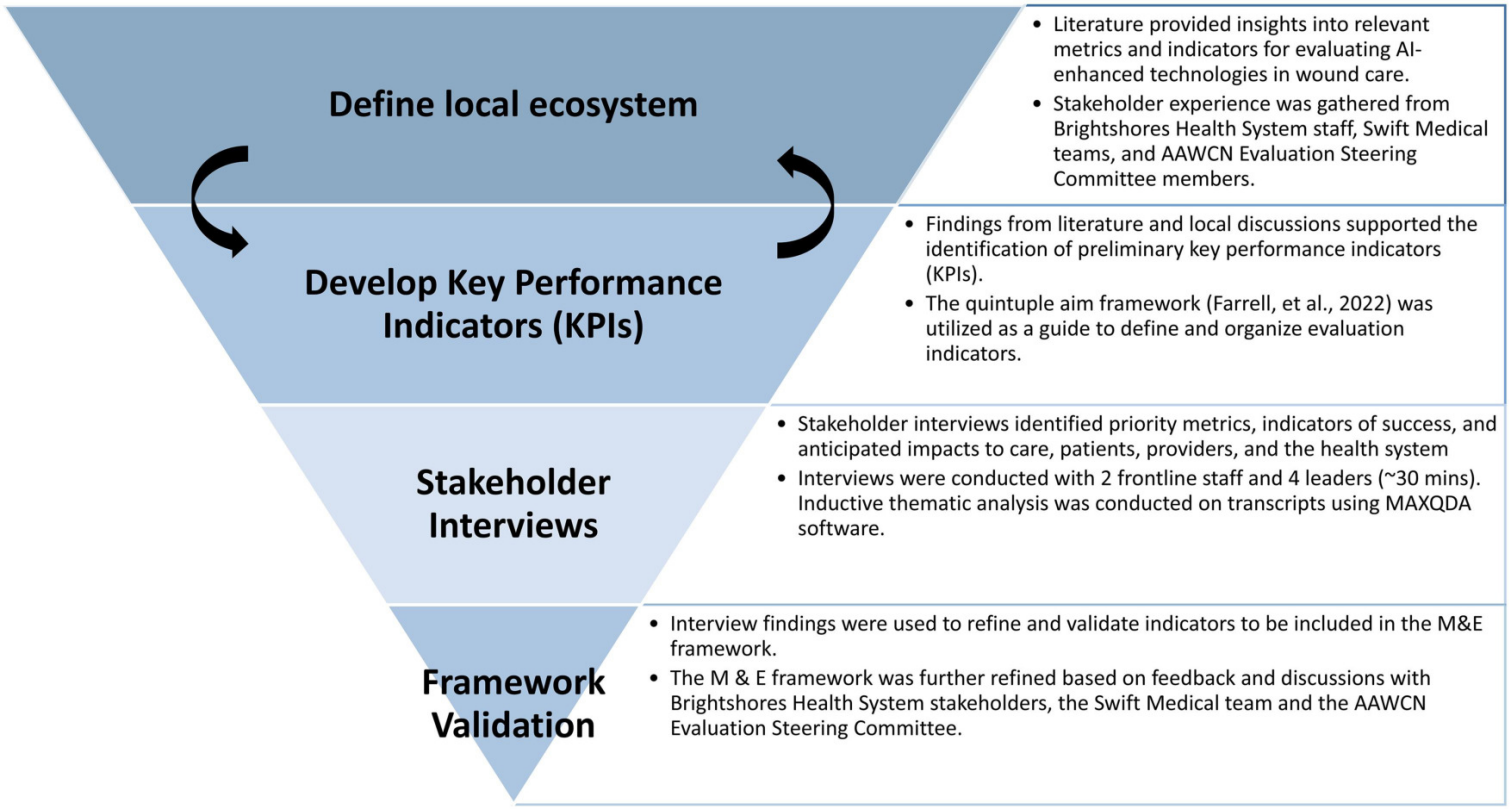
Multi-step iterative process of defining the evaluation framework.

We conducted a qualitative study involving a literature scan, semi-structured interviews, and regular monthly meetings with AAWCN implementation partners to identify stakeholders’ value propositions, prioritise outcome measures and to identify barriers to AI implementation and sustainability at Brightshores (See Figure 2). These activities were reviewed by the Research Ethics Committees at Trillium Health Partners, Institute Better Health (Study ID #239). Administrative approval for research ethics exemption was also received from the Brightshores Health System Research Advisory Committee (Study ID #110).

### 2.1 Literature scan

We conducted a purposive literature scan to identify commonly used and reported metrics and indicators on wound assessment, healing monitoring, and care outcomes in the field of wound care generally and more specifically using digital tools.

Publications and reports were identified by running an open search in February 2024 using PubMed and Google Scholar. These two databases were selected to capture both peer-reviewed literature and grey literature (e.g., reports, implementation procedures), which are particularly relevant in applied digital health contexts. Iterative combinations with Boolean operators (AND, OR) of the following search terms) were used: “wound care,” “digital health or artificial intelligence,” and “evaluation.” Our non-exhaustive search prioritised papers and reports that incorporated digital health interventions (DHI) or AI in the assessment and management of wounds. However, we did not restrict sampling to only DHI/AI-mediated studies. For example, we included papers that reported specific data collection tools and surveys for wound care such as WOUND-Q^©^- an internationally validated patient-reported outcome measure for research and clinical practice (25). The purpose of the literature scan was not to conduct a systematic synthesis and comprehensively identify all possible metrics or evidence on wound care evaluation, which has been done by other researchers (7, 26, 27). Rather, we aimed to compile a broad list of commonly reported indicators and metrics applied in real-world evaluation contexts, with a focus on empirical and practice-oriented sources. This was to serve as a pragmatic starting point for the development of a draft Monitoring and Evaluation (M&E) framework. In addition, this list informed probing questions during subsequent stakeholder interviews, and informed implementation planning. First, articles were screened based on titles and abstracts (or in the case of reports, executive summaries) to assess relevance. Second, full texts were reviewed for studies that described, evaluated, or utilized measurable indicators related to wound care processes or outcomes.

A target of 20 full-text articles was established *a priori* as a pragmatic sample size informed by rapid and applied evidence synthesis methods (28–30). This threshold was informed by feasibility considerations and the expectation that from these, we would likely capture the most commonly used metrics, whilst leaving room for additional ones to emerge during implementation partner meetings. Full texts were reviewed to extract key metrics and measures related to wound care practices and outcomes. A total of 17 papers were identified and screened for full-text review.

### 2.2 Implementation partner meetings

The list of metrics and indicators of wound care experience and outcomes identified through the literature scan were tabulated into an Excel sheet alongside the outcomes already identified in the Swift-AI implementation logic model (Figure 1). The table was presented to members of the AAWCN involved in implementation, including clinical and organisational leaders (e.g., Vice President of Clinical Innovation, Director of Health Services, Director of Research and Innovation), project managers, academic leaders, and program advisors, who met monthly to share feedback and experiences related to the implementation of the Swift Skin and Wound tool across sites. During these meetings, consortium members engaged in discussions to assess the relevance of each metric and the feasibility of data collection, including considerations related to data access, frequency of collection and most responsible party. Metrics that were not considered relevant to the project or viable for tracking (such as adherence to therapy, mortality rate, tissue oxygenation), were excluded (see Results and Supplementary File S1). Consortium partners also proposed additional metrics and indicators as measures of intervention success. Drawing on their understanding of the implementation context within their respective settings, as well as the capabilities of the technology, input from partners outside the Brightshores implementation team, including Swift Medical Inc., Giishkaandago'Ikwe Health Services, the National Research Council of Canada Aging in Place program, and the Centre for Technology Adoption for Aging in the North at the University of Northern British Columbia, informed the final list of key performance indicators (KPIs).

### 2.3 Semi-structured interviews

To ensure the evaluation would capture data that was relevant to clinical and investment priorities of decision makers within Brightshores, six semi-structured interviews were conducted prior to Swift Skin and Wound implementation over a 2-month period (April–May 2024) with a sample of four senior leaders from the hospital and two frontline staff at the Owen Sound DFU clinic, who represent primary end-users of the Swift Skin and Wound technology. Individual interviews were selected over a focus group approach to enable candid discussion across hierarchical roles and to elicit diverse perspectives relevant to implementation and evaluation planning. These interviews focused on the project's alignment with Brightshores' strategic goals, potential challenges in scaling and sustaining the technology, and anticipated impacts of Swift Skin and Wound on clinical practice. The decision to interview frontline staff at the DFU clinic, one of the six planned implementation sites, was guided by its role as the referral hub for chronic diabetes-related wounds across the health system, and the location from which the projects' implementation champion primarily operates from. It was thus considered a key location for gathering early insights into the technology adoption and integration process.

Interviewees were purposively selected based on their involvement in, or knowledge of the planned implementation of Swift Skin and Wound and awareness of wound care. At the time of the interviews, participants were provided with a general description of the tool's intended purpose and capabilities, but had no hands-on experience with it. Selected respondents were invited via email to participate in a virtual or in-person interview scheduled at a time convenient for them. Interviews were conducted by a researcher from the Brightshores Office of Research and Innovation, who is experienced in qualitative research. Interviews lasted approximately 30–45 min and were audio-recorded following participant consent. All interviews were transcribed and reviewed for accuracy.

### 2.4 Data extraction and analysis

The metrics and indicators for wound assessment identified from the literature were extracted into an Excel spreadsheet. In addition to a short description of each article, we extracted the following information: (i) data collection tools and data sources for monitoring and evaluating outcomes; (ii) outcome indicators reported in each paper categorised against the quintuple aim- clinical/population health, patient experience, provider experience, cost and health equity (31). The quintuple aim was selected as a reference point for outcome measures as it has become a commonly adopted lens for describing healthcare goals in Canada and globally (31).

Discussions and feedback from the monthly AAWCN consortium meetings were documented through detailed meeting minutes, which were reviewed by the research team and used to inform successive refinements of the M&E framework. Updated versions were subsequently circulated via email to the implementation partners for further input and validation.

For the interviews, thematic analysis was conducted by two research team members using MAXQDA^©^ software. Analysis involved an iterative process of coding, categorizing themes, and interpreting the data through discussions with team members from Brightshores Office of Research & Innovation. We employed inductive and deductive coding to identify themes relevant to study objectives. First, codes were developed directly from participant responses without preconceived categories, following which we assessed if the data aligned with the pre-defined themes explored in the interview guides. From these, a comprehensive codebook was defined.

## 3 Results

### 3.1 Draft monitoring and evaluation framework

From the literature scan, we identified a total of 14 articles and reports (see Supplementary File S1) of which only six focused on DHI-mediated wound care (32–37). Data collection tools included wound assessment forms, extracts from electronic health records/chart review, questionnaires, clinical measurements and risk score calculations. Most papers focused on outcomes related to two domains of the quintuple aim—population health and clinical outcomes, with metrics such as time to healing, occurrence of complications, knowledge attitudes and practices, infection rates and frequency of dressing. None of the papers captured equity measures, except if inferred through the collection of sociodemographic data (37). Though not widely tracked, cost-related outcomes were defined by hospitalization and transportation-to-care costs (33, 36), and savings from reduced medical supplies (38). The WOUND-Q (25) had the most robust compilation of patient experience measures for wound care. Through regular review and consultation meetings with implementation partners, KPIs that could be relevant to the AAWCN project were selected for subsequent validation. Understanding the current state of service delivery and the implementation ecosystem at Brightshores (i.e., a rural healthcare network) helped inform the draft M&E framework.

### 3.2 Qualitative results

Three overarching themes emerged from interview analysis reflecting (i) expectations of Swift Medical's wound care technology; (ii) considerations for implementation and evaluation; and (iii) potential barriers to adoption and implementation.

#### 3.2.1 Expectations from swift medical's wound care technology

Key informants revealed a strong alignment between the adoption of Swift Medical's wound technology and the strategic goals of Brightshores, particularly in its aim to be positioned as a leader in system-wide innovations to improve patient care in rural Ontario. Stakeholders described their hopes for Swift Skin and Wound to enhance clinical efficiency and improve patient outcomes and also saw it as an opportunity to address longstanding health inequities experienced by geographically marginalised populations across the health system. The following section captures key findings on expectations regarding the adoption of AI for wound care.

##### 3.2.1.1 Improve and standardise documentation

Overall, interview participants shared optimism that Swift Skin and Wound could significantly improve the standard of wound care at Brightshores by addressing longstanding variability in assessment and documentation practices. Clinical staff viewed its adoption as an opportunity to standardise wound assessments, improve accuracy in tracking healing trajectories overtime, and strengthen consistency of documentation. By supporting reliable longitudinal record of wound healing, the technology was seen as a way to reduce the risk of missing important clinical details and enhance reliability of patient information.

“[wounds] don't always get measured, even though they should every visit. So, standardizing that and making sure we have a measurement, we’ve got tissue quality, that sort of thing. I think is already going to bring our standard of care up.” [Frontline Staff]

Wound photos and automated measurements and descriptors, such as wound size, tissue characteristics, and healing trajectory were seen as beneficial outputs to reducing variability and inconsistency between provider assessments.

“So, in terms of impact, I can see just from a clinical perspective, having multiple different people looking at the same wound day to day, there's always that variability in interpretation. I can see a huge benefit in having sort of a quantitative perspective or the same software looking at it every day and getting the same information because then you get a much more accurate picture of what's actually happening with the wound.” [Health System Leader]

For mobile providers like the Nurse Wound Specialist who travels between the six networked sites, having the platform available on mobile devices such as on an iPad rather than a desktop was seen as more practical and effective for mobile wound care. Furthermore, Swift Skin and Wound was seen as a practical tool to enhance continuity of care as it enables other staff who may not necessarily be wound care specialists to also support wound care and follow-up.

“I'm very excited about that because it now creates like a flip book, essentially over time, it will create the potential for other people to take pictures of that wound so that instead of me getting to see that person once a month, now I have a picture of it once a week.” [Frontline Staff]

##### 3.2.1.2 Support clinical decision making

Participants similarly anticipated that Swift Skin and Wound would enhance clinical decision making by reducing subjective variation in wound assessments. The use of standardised images and metrics was seen as a way to improve clarity and consistency while enhancing communication among clinicians.

“Everybody's subjective assessment skills are different and what you write and describe might not actually be what you're trying to say. And so if you have a picture there to back up what you're saying, then it's a lot easier for somebody else to understand.” [Frontline Staff]

Health system leaders echoed this sentiment, emphasizing the value of having a shared visual reference to support more accurate and standardised evaluations, moving away from reliance on manual or purely visual assessments.

“It'll be better to identify the wounds and the significant stage of the wounds through the AI technology rather than somebody looking at it and making that assessment…the accuracy of using the technology is going to be a benefit.” [Health System Leader]

Leaders also saw potential for Swift Skin and Wound to enable data-informed care planning, utilizing the technology's metrics on wound severity, healing rates, and treatment timelines to identify system gaps and inform quality improvement efforts.

“If we find out that our stage 3's are taking longer than others to heal, it might help point us to what we need to [improve] for people with, certain stage or certain size or wound location” [Health System Leader]

In addition to its ability to improve assessment accuracy, Swift Skin and Wound was expected to support more efficient triage and care prioritization.

##### 3.2.1.3 Improve care navigation and quality of care

Beyond documentation, frontline staff also envisioned that the use of AI in wound care would support real-time monitoring of patient progress, from referral to discharge, replacing the need for manual chart audits and improving visibility into patient outcomes, as one interviewee captured the current state:

“right now there's no way to track whether other than me writing it down, if somebody has successfully been discharged and their wound has healed or how often they are ending up back in our clinic…you have to do like a chart audit and go through all the notes to see if they've ever been readmitted.” [Frontline Staff]

Staff further anticipated that earlier detection and consistent monitoring would reduce the incidence and severity of chronic wounds and pressure injuries, leading to overall system-level benefits such as shorter hospital stays and cost savings:

“Having it in the hands eventually of frontline staff will reduce the number of missed wounds and pressure injuries and reduce the number of people with pressure injuries that escalate.” [Frontline Staff]

“We will be able to catch them right where they start or prevent them from deteriorating further while they're here which is going to help with our length of stays. It will help with our financial costs and that sort of thing.” [Frontline Staff]

For patients with complex or chronic wounds, such as diabetes-related foot ulcers or pressure injuries, Swift Skin and Wound was expected to improve access to appropriate care by reducing delays in referral and treatment.

“If we can accurately measure and determine the tissue of the wound bed itself, and grade it as far as the ulcer is, then we can ensure they're getting referred to the right people at the right time, and ultimately improve their care, and it won't waste their time by sending them somewhere they don't need to go. So just making better use of the patient's time and improving their access to the right care at the right time.” [Health System Leader]

Staff also viewed Swift Skin and Wound as a tool for identifying patients who are not healing as expected, prompting earlier intervention which could potentially reduce follow-up visits and length of stay.

“I think [Swift Skin and Wound] will certainly help to guide treatment and help us to intervene sooner. People who are not making progress…my hope is that success will look like fewer return visits in the clinic and people not having such a long length of stay with us.” [Frontline Staff]

Participants anticipated that the adoption of Swift Skin and Wound would further enhance patient care by fostering greater engagement if patients could visualise their own healing progress, they could be motivated and more involved in self-management. Staff described visual data as a powerful way to strengthen communication and help patients understand their treatment journey and build confidence in the care that they receive.

“Patients love to see that things are progressing… they always [ask] me, ‘is it smaller than it was last week?’…a picture is worth a thousand words.” [Frontline Staff]

##### 3.2.1.4 Improve team based care and reduce workload

Stakeholders consistently identified the need to strengthen collaboration and communication across care teams at Brightshores. Frontline staff pointed to the opportunity to address the lack of a centralised system:

“I'm hoping it will improve the communication. I think that's the biggest challenge for us in the current environment…I'm hoping that everybody has access to this dashboard and can follow along the patient journey.” [Frontline Staff]

Interviewees also emphasised that the technology could shift how responsibilities are currently distributed. For example, by equipping frontline nurses with tools to assess and manage lower-priority wounds, the sole Nurse Wound Specialist who is currently tasked with validating all wound care decisions could focus on more complex cases, reducing burnout and improving resource allocation.

“We can offload some of those lower priority cases and simpler cases to the bedside nursing staff so we would be able to ensure that our wound care specialist is truly just seeing the more complicated cases.” [Health System Leader]

Staff anticipated that Swift Skin and Wound would enable a more coordinated care environment for all providers to engage in aligned care planning and ultimately improve patient experience.

“I think it can benefit [our Nurse Wound Specialist] and not having to maybe be everything to everybody because right now we don't have enough resources in that particular area.” [Health System Leader]

“Just making sure that we all have a platform in which we can communicate a little bit more transparently… would definitely be an outcome indicator.” [Health System Leader]

#### 3.2.2 Considerations for implementation

When asked about what would support successful integration of Swift Skin and Wound into clinical workflows, interview participants identified several factors including tailored training, strong leadership, peer engagement, and realistic workflow planning. These were seen as essential components of effective implementation and long-term sustainability of the technology.

##### 3.2.2.1 Education, resources, and training

Given the novelty of the Swift Skin and Wound technology, interview participants emphasised the need for tailored education and training for clinicians across the six implementation sites. While most felt confident that staff could quickly grasp the basic functions of the technology (i.e., taking pictures of wounds), they stressed the importance of continuous technical support including accessible resources such as step-by-step guides and hands-on training sessions. However, limited staff capacity was cited as a significant barrier to participation; many clinicians were already stretched thin, and carving out time to attend training sessions might be difficult, as one senior leader explained,

“One of the challenges right now is getting people to come to education events because everybody is short staffed… the clinicians will regularly set up lunch and learns for people to come learn about new things, but they can't get anybody to come…” [Health System Leader]

##### 3.2.2.2 Effective communication and leadership support

Participants underscored the need for early and transparent communication to clinical teams about the purpose and value of the Swift Skin and Wound implementation pilot. Building awareness ahead of implementation was seen as essential for addressing initial questions, supporting learning, and identifying “super users” who could guide adoption at the unit level. It was recommended that co-developing the implementation and adoption plan with frontline staff would ensure alignment with their clinical realities.

Sustained engagement from hospital leadership was also noted as a driver of momentum beyond the initial adoption period. Participants shared that visible support from administrative leaders in how the tool was being used, such as through participating in regular huddles and demonstrating active interest, would reinforce frontline engagement and buy-in at the bedside.

“I think it's important to keep our eye on how they're utilizing it as well… if they're using it clinically, then they'll ask for it to be used more… The opposite is also true if they completely disregard it…” [Health System Leader]

Identifying and empowering early adopters was also mentioned as a strategy for sustaining enthusiasm and building trust and confidence in using the technology. Participants proposed that nurse practitioners, coordinators, and other informal leaders could serve as “champions” to mentor peers and provide ongoing support, troubleshoot issues, and promote best practices throughout implementation. Engaging skeptics was also proposed as a method to address potential resistance to technology uptake, particularly with respect to workflow disruptions or concerns about using AI in clinical practice.

##### 3.2.2.3 Change management

All participants acknowledged that implementing new and sophisticated technology, while promising, would temporarily disrupt existing workflows and organizational structures. The importance of clearly communicating this “period of adjustment” was necessary to foster transparency, normalise short-term challenges, and support staff buy-in:

“Everyone wants to know why they're doing something or why am I spending my effort and energy doing this?” [Health System Leader]

One anticipated implementation adjustment was temporary reduction of patient load during the rollout phase to accommodate the time required for wound assessments and to complete documentation tasks—activities not currently embedded in routine care. Open conversations about these trade-offs were suggested as crucial for managing expectations and socializing the long-term benefits of standardised wound documentation.

“It's going to slow us down more because it's going to take longer to do these assessments because that's not something we're doing right now. But the charting will be more thorough, and I think will ultimately be a benefit, but it will take more time in the immediate short term” [Frontline Staff]

At the same time, participants stressed the need to balance staff workload to avoid burnout during implementation, while maintaining the quality and provision of patient care, emphasizing that new tools must enhance, and not hinder, clinical processes. Reflecting on past experiences, some respondents warned that if new initiatives are not perceived as valuable to patient care, they are likely to be abandoned.

“We’ve rolled out other scales in the past where the value or the number at the end wasn't meaningful…if the value or the product at the end of it isn't going to be utilised for the care of the patient, then it all of a sudden becomes a waste of time for everybody, regardless of how nice it is and how easy it is to implement and everything” [Health System Leader]

To minimise implementation resistance, leaders advised integrating evaluation data collection into existing workflows and avoiding manual processes that could compromise data quality or further increase staff workload.

“Try and pick indicators that are easily obtainable without asking somebody to do extra work, because as soon as you start saying you need to collect this manually, then I think the integrity of the data suffers” [Health System Leader]

#### 3.2.3 Potential barriers to adoption and implementation

While interview participants expressed strong enthusiasm for the potential of Swift Skin and Wound, they also shared concerns about the practical realities of implementation, particularly in a rural health system already faced with resource constraints, staffing pressures, and challenges with digital infrastructure. The following themes reflect anticipated barriers that could limit uptake, stall integration, or affect potential long-term sustainability of the innovation.

##### 3.2.3.1 Usability and integration

One of the anticipated challenges identified by staff was ensuring that Swift Skin and Wound integrates seamlessly with Brightshores' existing electronic medical record system, Cerner Oracle. Participants emphasised that unless the technology could be embedded directly into existing documentation workflows, its long-term usability may be compromised, creating inefficiencies and discouraging frontline adoption. If the project were to extend beyond the pilot phase, the ability to link data from wound assessment with large health administrative datasets and support longitudinal tracking of patients was noted as an advantage.

“I think that one of the barriers is going to be integrating it seamlessly with our current electronic medical record…if we're going forward with this beyond the two-year pilot, I would want to make sure that we can fully integrate it.” [Health System Leader]

“While we won't be able to fully integrate it into Cerner, we hope that the information is readily accessible. for clinicians and can still facilitate some of that communication and information sharing in maybe an easy and standardised way.” [Health System Leader]

##### 3.2.3.2 Increased workload and burden

Although the introduction of Swift Skin and Wound was expected to distribute wound assessment responsibilities across frontline staff, interview participants anticipated that staff could perceive introduction of the tool as an addition to their workload.

“If one wound assessment takes 12 min, and then we're introducing something that's going to take 45 min, it's a big difference in the amount of time… they'll recognise that it is best practice but they may anticipate some pushback \if there's a significant or perceived significant increase in their workload.” [Health System Leader]

Beyond staff-level concerns, respondents also flagged the potential unintended consequence of increased reliance on specialised staff, particularly the sole Nurse Wound Specialist. While decentralizing wound assessments could enhance documentation and responsiveness, leaders cautioned that without clear protocols and redistribution of responsibilities, the Nurse Wound Specialist may still be expected to validate every assessment completed by other staff to ensure consistency and quality of documentation. Stakeholders voiced that this additional step could inadvertently centralise responsibility rather than distribute it, reinforcing unsustainable pressure on one individual which could potentially compromise the long-term success of the pilot.

“Without extra resources and with putting up too much on one person, that's a single point weakness and if we lose that expert then the project is in jeopardy.” [Health System Leader]

Participants emphasised that addressing this barrier would require shared accountability, streamlined triaging protocols, and empowering frontline staff to independently manage low-complexity wounds supported by the AI technology. This would ensure that the Nurse Wound Specialist remains focused on complex cases, preventing bottlenecks and protecting the sustainability of the initiative.

##### 3.2.3.3 Impacts on patient care

While stakeholders were optimistic about the potential of Swift Skin and Wound to improve wound care documentation, they flagged anticipated impacts on patient care that could arise early during implementation. In a health system already stretched thin, participants expressed concern that introducing a new technology could temporarily disrupt clinical efficiency, patient flow, and further strain staff capacity. Until clinical workflows are adapted, patients, more specifically at the DFU clinic may experience longer wait times, and staff may feel pressured to balance quality with efficiency.

“We may need to look at how we book our appointments the first couple of days until we can figure out the timing and how that's going to be impacted by the new program.” [Health System Leader]

Participants also noted that new documentation procedures such as the addition of image capture requirements may introduce clerical and logistical demands for staff. Stakeholders drew from previous digital implementation efforts, such as the introduction of iPads during the pandemic, to emphasise the importance of process-level details. Issues like locating, charging, or sharing devices can become major points of friction if not addressed early on.

“it's more of a process piece to sort of say, where is the iPad when you need it?…Can you standardise the location of it so that it's always there so that when you go looking for it…it's like making sure there's a dedicated spot for and it's always there and then always charged.” [Health System Leader]

It was noted that additional support may be needed to prevent these operational changes from adversely impacting patient experience. Ensuring that devices are readily and consistently accessible and seamlessly integrated into clinical routines was seen as essential to maintaining patient care quality during the pilot phase.

Finally, participants highlighted the importance of respectful and informed patient consent when using Swift Skin and Wound. While most patients are expected to accept the use of wound images as part of their care, some individuals and certain populations may require additional sensitivity. Clear, consistent communication and consent protocols was mentioned as being necessary to uphold patient trust and privacy.

“I already say to patients about why I'm taking a picture, where it goes, and their confidentiality. I think we need to ensure that we are getting proper consent and probably signed consent for these photographs into their charts. Most often I just get a verbal consent, and I'm not sending the pictures anywhere. They're just there for me to look at. But I think that's a really important piece.” [Frontline Staff]

##### 3.2.3.4 Financial considerations

Sustainability and scale-up of Swift Skin and Wound beyond the pilot phase was widely seen as contingent on a clear and demonstrable value proposition. The financial implications of adopting the technology across Brightshores Health System were noted as an important consideration for long-term decision making.

Senior leaders emphasised that the ability to demonstrate cost savings, particularly on wound care supplies, would be essential for building a strong business case. Equally important were the anticipated downstream benefits of improved wound documentation such as reduced wound-related hospitalizations, shorter lengths of stay, increased patient satisfaction, and enhanced quality of life. These non-financial outcomes were viewed as equally important indicators of value that would strengthen the case for long-term investment.

“To be able to say it made a difference because I think obviously patient outcome and finances always drive things too… what's the financial aspect of the program? Does it outweigh increased length of stay, patient outcomes, patient satisfaction, the supplies that we're using for big wounds?” [Health System Leader]

While some leaders viewed the upfront cost of the technology as secondary to its overall impact on patient care, others stressed that the procurement decision of Swift Skin and Wound would ultimately hinge on alignment with budgetary and organizational priorities. Stakeholders underscored that presenting a balanced case, demonstrating both financial savings and clinical benefits, would be essential to securing leadership support for scaling and sustaining Swift Skin and Wound beyond the pilot period. Table 1 below summarises the key enablers and barriers to implementation within the context of Brightshores.

**Table 1:** Key enablers and barriers to implementation.

Domain	Enablers	Barriers
Clinical practice, decision-making and quality of care *(population health outcomes)*	Standardised wound assessment and documentation practices Improved assessment accuracy and consistency across providers through shared image-based references Longitudinal tracking of wound healing trajectories enabling earlier identification of non-healing wounds Data-informed clinical decision-making and care planning	Initial increase in assessment time Learning curve associated with new wound assessment procedures and clinical workflows Risk of over-reliance on specialist validation without clear protocols
Team-based care, leadership and change management *(provider experience)*	Redistribution of wound care responsibilities, reducing burden on sole Nurse Wound Specialist Improved provider communication and coordination through shared dashboard Strong leadership and hands-on support, identification of champions and early adopters Clearly communicated value proposition	Workforce shortages and limited time and capacity for (re)training Risk of sustaining centralized responsibility on sole Nurse Wound Specialist if roles and share accountability across clinical staff are not clearly defined Adoption resistance if perceived value is unclear to staff and patients
Care navigation and patient experience *(patient experience and equity)*	Improved visibility of patient trajectory from referral to discharge Timely and more appropriate referrals Enhanced patient engagement through visualisation of healing progress	Temporary disruption to clinical flow and appointment scheduling during early rollout Need for consistent consent process for visual wound capture Safeguarding privacy and trust in AI-enabled wound assessments, with considerations towards image capture, data governance, and digital data security, particularly for vulnerable populations
Operational workflow and financial costs *(cost implications)*	Long-term efficiency gains through standardisation Reduction in manual documentation and audits Potential cost savings from reduced escalations and hospitalisations	Need for demonstratable value to justify long-term investment Budgetary constraints and competing organisational priorities
Digital infrastructure and integration	Mobile accessibility supporting care providers across multiple sites System-wide data visibility and accessibility	Limited interoperability with existing EMR system Risk of duplication and increased documentation burden

### 3.3 Validation of the final monitoring and evaluation framework for the AAWCN project

Findings from the literature scan, stakeholder interviews, and implementation partner meetings were triangulated to inform the final version of the M&E framework. Each data source contributed distinct but complementary insights; the literature scan provided a foundational set of commonly used and validated metrics; interviews contributed contextual and experiential insights related to the wound care context at Brightshores, and consortium discussions supported feasibility assessment and refinement of indicators for implementation and evaluation. Together, these data sources informed the identification, refinement, and validation of key metrics and measures for the implementation and evaluation of the Swift Skin and Wound tool.

Population health and clinical outcome and cost-related measures were primarily informed by the literature scan, which primarily identified quantitative indicators such as frequency of patient visits, number and type of wounds, healing time, hospitalization, amputations, health service utilization, and costs associated with wound care per patient. In contrast, technology-related metrics such as system reliability and accuracy, user satisfaction, and cost analyses were guided by the Swift Medical team and refined through implementation partner meetings with the input of health decision makers at Brightshores.

Equity-related measures were not thoroughly represented in the literature scan and were therefore largely informed by the experiential knowledge of the implementation team at Brightshores and interview participants. These perspectives emphasized the importance of capturing equity-relevant indicators, including the proportion of patients experiencing marginalisation, using the Ontario Marginalization Index. This index is a validated, regional-based measure that captures dimensions of marginalisation informed by indicators derived from sociodemographic data including age, labour force participation, socioeconomic status, housing characteristics, and racialisation.

Interview findings further highlighted key considerations for successful adoption and implementation of Swift Skin and Wound. This included context-specific metrics such as distance travelled, travel time, administrative burden, clinician confidence in wound assessment, and ability to identify wounds. All of these reflected the clinical realities, documentation practices, and care delivery context at Brightshores. These insights were integrated with metrics identified through the literature scan and further refined through iterative discussions with the AAWCN consortium. Follow-up discussions involved refining metric definitions for completeness and clarity, validating the feasibility of data collection from technical and operational angles, and aligning implementation partner and project stakeholders on appropriate timing for phased data collection to minimise monitoring fatigue.

For instance, within the quintuple aim domain of Population Health/Clinical Outcomes, stakeholders identified time to wound closure as a key outcome to be assessed at endpoint. This metric will be evaluated using measurements captured through the Swift Skin and Wound platform at each clinical encounter, supplemented by EMR data where wound closure date is documented. Tracking this outcome will enable clinicians and decision-makers assess changes in healing trajectories over time, compare rate of wound healing before and after implementation, and assess whether the technology contributes to more timely wound resolution within the local context. The final version of the M&E Framework is presented in Table 2.

**Table 2:** Revised and final monitoring and evaluation framework.

Quintuple aim domains	Specific indicators/Metrics	Data collection information			
		Time period			Methods
		Baseline	Midline	Endline	
Patient experience					
	Frequency of visits of same patient			✓	Electronic medical record (EMR) data extraction
	Total number of assessments		✓	✓	EMR data extraction
	Total number of unique patients monitored with technology	✓	✓	✓	Swift AI data output
	Distance travelled/time to reach service				Patient residence postal code matched to hospital site
	Patient engagement in care plan	✓	✓	✓	Patient experience survey (swift template) and/or qualitative interviews
Logic model outcomes	Patient satisfaction with wound care management	✓	✓	✓	Patient experience survey (swift template) and/or qualitative interviews
	Communication with patients and their engagement in tracking wound progress	✓	✓	✓	Qualitative interviews
	Use of the AI application to conduct a standardised wound care evaluation.	✓	✓	✓	Swift AI data output
Provider experience					
	Clinician travel time	✓	✓	✓	Calculated manually from nurse wound specialist logs
	Clinician confidence in clinical decision re: wound assessment and management	✓	✓	✓	Qualitative interviews and/or staff survey
	Clinician satisfaction with technology			✓	Staff survey
	Administrative burden	✓	✓	✓	Qualitative interviews and/or staff survey
	Frequency of visits of same patient	✓	✓	✓	Swift AI data output
Logic model outcomes	Patient satisfaction with wound care management	✓	✓	✓	Qualitative interviews and/or staff survey
	Communication with patients and their engagement in tracking wound progress	✓	✓		Qualitative interviews
	Use of the AI application to conduct a standardised wound care evaluation			✓	Swift AI data output
Cost implications					
	Average time to discharge			✓	EMR last date of visit minus first date of visit (assumption)
	$ value per patient served			✓	Costing analysis by swift
Logic model outcomes	Reduction in Brightshores health service utilization (reduced # outpatient visits, reduced hospitalizations)			✓	Costing analysis by swift
	Healthcare costs associated with wound care management			✓	Costing analysis by swift
Population health/clinical outcomes					
	Referral time	✓		✓	Calculated manually from paper charts
	Frequency of visits of same patient	✓		✓	EMR data extraction
	Frequency of visits of same patient	✓	✓	✓	Swift AI data output
	Number of wound referrals			✓	EMR data extraction
	Number of patients discharged (DFU)	✓	✓	✓	EMR data extraction
	Completeness of wound documentation	✓		✓	EMR data extraction
	Patient wound prioritization (decision-making)	✓	✓	✓	Swift AI data output
	Inter/intra-reliability of autodepth and smart tissue	✓	✓	✓	Swift AI data output
	Number of wounds assessments/encounters completed (by site, dept)			✓	EMR data extraction
	Number of wound assessments (Swift PDF) shared from Brightshores to community			✓	TBD
Logic model outcomes	Incidence of in-house acquired wounds			✓	EMR data extraction
	Time to wound closure			✓	Swift AI data output; EMR data extraction
	Number of amputations of lower limb		✓	✓	EMR data extraction
	Number of surgical debridement of lower limb		✓	✓	EMR data extraction
	Number of advanced DFU procedures		✓	✓	TBD
	Hospitalization rate and ER visits due to wound-related issues			✓	EMR data extraction
Equity					
	Identify & define underrepresented populations that technology is applied to	✓	✓	✓	ON-Marg index analysis & EMR data extraction
	Visual accuracy rates of AI features for older skin underrepresented populations vs. other populations	✓	✓	✓	Swift AI data output
	Patient inclusion in clinical decision-making	✓	✓	✓	Qualitative interviews and/or patient experience survey (swift template)
	Number of patients that are marginalised				compare residence postal code with hospital location—GIS
	Identify & define underrepresented populations that technology is applied to (elderly skin, lower socioeconomic status clients)	✓	✓	✓	^a^ON-Marg index analysis
	Age disaggregated service uptake and outcome				ON-Marg index analysis
Logic model outcomes	Quality of wound care across diverse populations (considering age, ethnicity, and gender)	✓	✓	✓	EMR data extraction
Other					
	Number of clients receiving IV antibiotics for wound infection	✓	✓	✓	EMR data extraction
	Better identification/recognition of in-house acquired wounds		✓	✓	EMR data extraction

a Ontario marginalization index (ON-Marg): a population-level tool that measures dimensions of geographic marginalization (e.g., household and dwellings, material resources, age and labour force, racialised and newcomer populations) across Ontario (39).

## 4 Discussion

### 4.1 Principal findings

This study developed and validated a monitoring and evaluation framework to guide the implementation and evaluation of Swift Medical's AI-enabled wound care technology within a rural healthcare network, Brightshores Health System. By gathering perspectives from frontline staff, healthcare leaders, and implementation partners through a rapid literature scan, stakeholder interviews, and regular consortium meetings, we identified expectations of the technology, key implementation considerations, and potential barriers to adoption and sustained use. The final M&E framework comprised 45 indicators linked to the quintuple aim including patient and provider experience, cost implications, population health and clinical outcomes, and equity-related considerations. Through iterative consultation and refinement, the framework was tailored to address context-specific challenges and priorities within Brightshores. Our findings highlight the complex interplay of clinical needs, organizational readiness, and technological integration necessary for successful AI deployment in a rural resource-constrained, multi-site health institution. By embedding diverse perspectives throughout the evaluation design process, this work offers a replicable model for developing responsive, stakeholder-driven evaluation frameworks that promote implementation ownership, guide endpoint decision-making, and enhance sustainability of AI-enabled healthcare innovations.

The development of the M&E framework offered key insights into how stakeholders defined “success” differently. Health system leaders valued demonstratable outcomes such as clinical measures and data on wound healing rates, patient satisfaction, clinician efficiency and cost-effectiveness, reflecting metrics that align closely to strategic and financial priorities. In contrast, frontline staff underscored workflow barriers linked to technical (e.g., data charting and interoperability of EMR systems, lack of documentation standards), individual (e.g., patients self-management) and interpersonal (e.g., culture of change and workflow within interdisciplinary teams) factors that shape wound care delivery at Brightshores. These concerns are within the context of a shortage of formal wound care expertise despite the region's high prevalence of chronic wounds (19, 40). Nevertheless, both frontline staff and system leaders expressed optimism that Swift Skin and Wound could significantly strengthen wound assessment accuracy and management, standardise care processes, and improve both patient and provider experiences if implemented with strong leadership and organizational support. While this initial phase prioritised the perspectives of frontline clinicians and health system leaders to inform implementation feasibility, governance, and sustainability of Swift Skin and Wound, patient and caregiver perspectives remain essential to comprehensive evaluation of AI-enabled wound care.

Study findings underscore the value of aligning evaluation with real-world implementation apriori, as seemingly minor operational issues, such as device storage and shared responsibility for maintenance, which interviewees identified as potential threats to adoption could be left unaddressed if not monitored for occurrence. These insights illustrate how overlooked operational details can undermine the effectiveness of high-value technologies. Given limited workforce capacity and burnout, all interviewees emphasised the need for low-burden, flexible, and just-in-time training integrated into existing workflows.

One of the issues identified by staff was the lack of structured and integrated documentation across care settings. Evidence has shown that inefficient documentation practices contribute to cognitive overload, burnout and hinder quality performance (41, 42). While Swift Skin and Wound is intended to reduce these pressures by standardizing wound assessments and documentation, study participants noted that long-term sustainability is largely dependent on the degree to which the tool can be integrated with existing EMR systems. Without such interoperability, even high-value AI-enabled tools risk underutilization due to duplicative efforts and increased administrative burden beyond the initial stage of tool familiarisation. Complimentary quality improvement initiatives, independent of new technologies, could deliver meaningful gains in documentation practices.

Unique equity considerations were particularly salient in Brightshores' rural and aging population, including Indigenous communities. While AI-enabled wound care holds promise, stakeholders noted that healing trajectories are shaped by patient engagement and social determinants of health (e.g., smoking, nutrition, housing, and socioeconomic status). These findings reinforce that AI-enabled innovations cannot achieve meaningful impact without addressing underlying social and structural drivers of inequity. Literature has cautioned that digital technologies and AI adoption have often benefited urban and socioeconomically privileged populations, risking widening disparities (43–45). Equity-related indicators should therefore remain central in evaluating AI-enabled wound care to ensure implementation strategies reflect local cultural and structural realities. Seven contextualised equity-related indictors are captured in the project's final evaluation plan.

Rural health systems present distinct challenges in the adoption of AI technologies in clinical settings, including workforce shortages and geographic barriers that exacerbate inequities in wound care (12). The M&E framework developed for the context of Brightshores prioritised indicators such as patient engagement, clinician confidence, documentation consistency, and equity factors. These reflect expectations that Swift Skin and Wound will not only enhance wound assessment but also reduce clinician burden, strengthen communication, and improve standards of care across sites. By capturing these dimensions, our aim is to ensure that evaluation reflects the realities of rural healthcare delivery and technology implementation. Incorporating patient and caregiver perspectives in future evaluation phases will be particularly important for understanding how AI-enabled wound care is experienced across diverse populations and for ensuring that implementation strategies do not inadvertently exacerbate existing inequities in access, trust, or engagement.

As implementation of Swift Skin and Wound progresses within Brightshores, the M&E framework will be applied to guide ongoing data collection, learning, and clinical decision-making. Future applications of this framework, locally and in other contexts, would benefit from validation and refinement of indicators through expanded stakeholder engagement to accommodate local priorities, data availability, and diverse clinical contexts. As AI-enabled technologies continue to evolve, evaluation strategies must remain contextually-sensitive and responsive to organizational needs to ensure sustained relevance and impact.

By engaging multiple stakeholders in evaluation framework development, this study ensured that evaluation indicators captured both frontline considerations and system-level strategic objectives. This collaborative process fostered ownership beyond financial investment and encouraged multi-stakeholder planning for adoption and implementation, surfacing contextual factors such as team culture and patient care-seeking behaviours, that ultimately shape AI adoption and readiness. These insights reinforce that evaluation is not a neutral exercise; rather, it must deliberately balance diverse perspectives to generate meaningful findings and sustain implementation. Ultimately, the utility of valuation findings is directly related to their ability to answer the questions decision-makers deem relevant- maximizing the likelihood of findings being actionable.

#### 4.1.1 Comparison to existing work

As evidenced by findings from the literature scan, existing evaluations of digital and AI-enabled wound care interventions have predominantly focused on clinical outcomes, such as healing rates, infection prevention, and time to closure, with less attention to patient experience, provider workload, and equity considerations (32, 34, 35, 37, 46, 47). By explicitly aligning indicators to the quintuple aim, our framework extends beyond clinical metrics to capture broader potential system-level impacts of AI adoption. This aligns with calls in the literature for more comprehensive evaluation approaches that consider the organizational, economic, and equity dimensions of digital health innovations (48–50). Importantly, the inclusion of equity-focused indicators, which are largely absent in prior wound care studies, addresses a critical gap, particularly given the rural implementation context and longstanding disparities in wound care access (10, 11).

### 4.2 Strengths and limitations

A core strength of this study is that the exploratory design and qualitative methodology provided rich insights into the expectations of health system leaders and frontline staff within Brightshores and their perspectives on the incorporation of AI-based technology such as Swift Skin and Wound. However, there are several limitations that should be acknowledged.

First, the literature informing draft framework development was derived from a purposive, non-exhaustive literature scan. This approach prioritised commonly reported and implementation-relevant metrics and indicators but may not have captured all possible DHI and wound care measures described in the literature. Second, the number of interview participants was relatively small. This limitation reflects the context of a rural hospital setting, where the pool of individuals involved in decision-making and implementation was inherently limited as staff often hold multiple roles and responsibilities across the organisation. However, consistent with a purposive sampling approach, participants were selected for their relevance and depth of expertise. Given the scope of inquiry, we prioritised information power which was sufficient to capture diverse perspectives (51). Furthermore, patient and caregiver perspectives were not directly included in this initial phase although patient experience and satisfaction metrics are embedded within the evaluation framework as captured through the literature scan and implementation partner meetings. Data collection during program evaluation can help surface additional patient and caregiver perspectives on metrics and outcomes that matter with AI-enabled wound care. Lastly, the framework was developed within the specific context of a rural, multi-site network, and while this setting offers valuable lessons, it also limits generalizability to urban or more resource-rich environments. These contextual and methodological constraints underscore the importance of viewing the proposed M&E Framework as adaptable and iterative, with the need for further adaptation and incorporation of additional stakeholder perspectives as implementation within Brightshores expands beyond the DFU, and if used in other health system settings.

## 5 Conclusion

Successful spread and scale of AI-enabled innovations require more than technological readiness. It is dependent on change management, stakeholder ownership, and deliberate evaluation planning. Without clarity on relevant metrics, and mechanisms for using findings to inform decision-making, interventions risk being adopted without evidence of meaningful impact or insights to guide sustainability.

Insights gained from this study demonstrate how engaging leaders and frontline staff in framework development ensures that evaluation reflects both strategy priorities and day-to-day realities of clinicians. As interest grows in deploying AI to improve equity and access in rural and remote contexts, the insights generated here reinforce the importance of tailoring evaluation approaches to local needs rather than applying generalised models. The M&E framework developed in this study can serve as a practical tool and adaptable reference point for similar initiatives. By measuring what matters, aligning investments with outcomes, and incorporating equity throughout, such frameworks can help bridge disparities in wound care access between rural and urban populations while guiding sustainable adoption of AI-enabled health innovations.

## Data Availability

The raw data supporting the conclusions of this article will be made available by the authors, without undue reservation.
