# Supplementary material for: Monitoring and evaluation of an artificial intelligence-enhanced wound care intervention in a rural health network: defining stakeholder expectations and shared priorities
Source: Front Digit Health. 2026 Jun 15;8:1774616. doi: 10.3389/fdgth.2026.1774616 (PMC13311118; doi:10.3389/fdgth.2026.1774616)
Supplement: Supplementary file 1 [file Table1.docx]

**Table S1**. Metrics and measures for evaluating wound care practices and wound care interventions identified through literature scan and refined through stakeholder consultation.

|  |  | **Quintuple Aim Outcomes** | | | | |
| --- | --- | --- | --- | --- | --- | --- |
| **Title of Paper/Report** | **Data Collection Tools** | **Patient Experience** | **Provider Experience** | **Cost Implications** | **Population Health/Clinical Outcomes** | **Equity** |
| Complex Wound Healing Outcomes for Outpatients Receiving Care via Telemedicine, Home Health, or Wound Clinic: A Randomized Controlled Trial^30^ | - Electronic Health Records (EHRs) - Telemedicine Platforms - Wound Assessment Forms - Questionnaires and surveys - Cost Tracking Tools - Mortality and Health Outcome Databases | - Healing Rate | None identified | - Transportation Costs | - Time to Healing - Mortality Rates | None identified |
| Wound management for the 21st century: combining effectiveness and efficiency.^49^ | None identified | - Prevention Strategies | None identified | - Economic cost | - Healing Time - Frequency of Dressing - Changes - Complications - Prevalence of Wounds | - Economic cost |
| Skin Wound Healing Process and New Emerging Technologies for Skin Wound Care and Regeneration. ^29^ | None identified | None identified | None identified | None identified | - Phases of Wound Healing - Complications in Wound Healing - Chronic Wound Care Treatments - Advanced Dressings | None identified |
| Managing wound care outcomes^50^ | - Retrospective Chart Review - Outcomes Management Model Framework - Cost Analysis Tools - Wound Assessment Tools | None identified | None identified | - Cost of wound care | - Wound Prevalence - Care Outcomes - Adherence to Best Practices | None identified |
| Wound Care Knowledge, Attitudes, and Practices and Mobile Health Technology Use in the Home Environment: Cross-Sectional Survey of Social Network Users.^31^ | - Four-part web-based questionnaire to gather information on participant demographics, wound care experience, wound care competence, and mHealth technology use related to wound care. | None identified | None identified | None identified | - Wound Care Knowledge - Wound Care Attitudes - Wound Care Practices | None identified |
| Expert Outpatient Burn Care in the Home Through Mobile Health Technology.^32^ | - Electronic medical records (EMR) - TeleBurn App. | None identified | None identified | None identified | - Wound Infections - Healing Time - Number of Clinical Encounters - Adherence to Therapy | None identified |
| Quality indicators for a community-based wound care centre: An integrative review.^51^ |  | - Patient engagement in wound care | None identified | None identified | - Effectiveness of nurse-led wound care services. - Implementation of evidence-based care and standardized care practices. | None identified |
| Real-world clinical evaluation and costs of telemedicine for chronic wound management.^33^ | - Medical software database called Infinys v1.8 (for clinical and demographic data) - National health insurance database (for economic data) - Patient files (for clinical outcome measures) | None identified | None identified | - Outpatient care costs - Travel costs - Hospitalizations | - Time to complete or partial healing - Percentage of wounds reaching target objective - Percentage of wounds healed completely | None identified |
| The Role of Telemedicine in Wound Care: A Review and Analysis of a Database of 5,795 Patients from a Mobile Wound-Healing Center in Languedoc-Roussillon, France. Plastic and Reconstructive Surgery.^34^ | - CICAT Medical database (Infynis) - Questionnaire (for telephone interviews with general practitioners) | None identified | None identified | None identified | - Types of wounds treated - Wound healing/improvement - Rate of unchanged or worsened wounds - Adherence to advice provided by CICAT | - Demographics |
| Developing a Practical Tool for Predicting Wound Healing Outcomes of Patients with Diabetic Forefoot Ulcers: Focus on Vasculopathy and Infection.^52^ | - Transcutaneous partial oxygen tension (TcPO2) measurements - Toe pressure measurements - Tissue and bone biopsy Cultures - Risk Score Calculations | None identified | None identified | None identified | - Vasculopathy - Infection rates - Wound healing outcomes (healed without amputation, minor amputation, and major amputation) | None identified |
| A Patient Navigation Model to Improve Complex Wound Care Outcomes.^35^ | - Clinical assessment reports - Electronic Health Records (EHRs) - Patient Surveys/Interviews: - Data analysis of healthcare utilization | None identified | - Reduced number of nursing visits | - Reduced usage of medical supplies | - Wound closure - Wound surface area reduction - Pain reduction | None identified |
| Patient-Centered Education in Wound Management: Improving Outcomes and Adherence.^43^ | Not specified | - Patient satisfaction - Self-management skills | None identified | None identified | - Patient Adherence - Wound healing outcomes | None identified |
| Evaluation of Surgical Wound Healing in Orthopedic Patients with Impaired Tissue Integrity According to Nursing Outcomes Classification.^44^ | Nursing Outcomes Classification Instrument | None identified | None identified | None identified | - Skin approximation - Wound drainage - Surrounding skin erythema - Peri-wound edema - Increased skin temperature - Foul wound odor | None identified |
| International study to develop the WOUND-Q patient-reported outcome measure for all types of chronic wounds.^25^ | - WOUND-Q scales | - Health-related quality of life (life, psychological, sleep and social impact) - Experience of care (information, home care nurses, medical team, and office staff) - Wound treatment (dressing and suction device) |  |  | - Wound characteristics (assessment, discharge, and smell) | None identified |
